# Time trend analysis of the prevalence and incidence of diagnosed type 2 diabetes among adults in Taiwan from 2000 to 2007: a population-based study

**DOI:** 10.1186/1471-2458-13-318

**Published:** 2013-04-09

**Authors:** Cheng-Chieh Lin, Chia-Ing Li, Chih-Yi Hsiao, Chiu-Shong Liu, Sing-Yu Yang, Cheng-Chun Lee, Tsai-Chung Li

**Affiliations:** 1 Department of Family Medicine, China Medical University Hospital, Taichung, Taiwan; 2 School of Medicine, College of Medicine, China Medical University, Taichung, Taiwan; 3 Department of Medical Research, China Medical University Hospital, Taichung, Taiwan; 4 Graduate Institute of Health Care Administration, College of Public Health, China Medical University, Taichung, Taiwan; 5 Graduate Institute of Biostatistics, College of Public Health, China Medical University, Taichung, Taiwan; 6 Department of Neurology, China Medical University Hospital, Taichung, Taiwan; 7 Department of Healthcare Administration, College of Health Science, Asia University, Taichung, Taiwan

**Keywords:** Type 2 diabetes, Prevalence, Incidence, Time trend analysis

## Abstract

**Background:**

The prevalence of type 2 diabetes has rapidly increased in the Taiwanese population with the increasing prevalence of a sedentary lifestyle and high-calorie dietary intake. This study aims to determine the annual trends of the prevalence and incidence of diagnosed type 2 diabetes among adults in Taiwan from 2000 to 2007.

**Methods:**

A population-based study of all residents aged 20 years and over (12,191,076 in 2000 and 18,772,180 in 2007) enrolled in the National Health Insurance (NHI) program, the database of which was used to identify patients diagnosed with type 2 diabetes. The annual prevalence and incidence of diagnosed type 2 diabetes were estimated using the International Classification of Diseases, 9^th^ Revision, Clinical Modification diagnostic codes based on age, gender, insurance premium, and urbanization degree. Logistic regression was used to estimate the odds ratios (OR) of risk factors, as well as to examine the trend in the annual prevalence or incidence of diagnosed type 2 diabetes from 2000 to 2007.

**Results:**

The crude annual prevalence of diagnosed type 2 diabetes increased significantly from 5.79% in 2000 to 8.30% in 2007. The increase was highest in 2007, among men, individuals aged ≥ 80 years, and individuals residing in aging society areas [OR (95% CI): 1.416 (1.412–1.420), 1.033 (1.032–1.034), 31.810 (31.690–31.931), and 1.090 (1.085–1.094), respectively]. The crude incidence fluctuated throughout the study period, ranging from 7.72 per 1,000 in 2006 to 8.98 per 1,000 in 2000. The decrease was highest in 2006, among individuals with an insurance premium ≥ median value [0.933 (0.925–0.942) and 0.810 (0.805–0.815), respectively]. The greatest increase was among men, individuals aged 60 to 79 years, and individuals residing in aging society areas [1.150 (1.145–1.155), 15.452 (15.329–15.576), and 1.127 (1.113–1.142), respectively].

**Conclusion:**

This study demonstrated the substantial increase in annual prevalence of diagnosed type 2 diabetes among adults in Taiwan from 2000 to 2007. The incidence fluctuated between 2000 and 2007.

## Background

Type 2 diabetes has rapidly increased in prevalence in Asian populations along with the increasing prevalence of a sedentary lifestyle and high-calorie dietary intake [1]. Diabetes develops at a younger age among Asian populations than among Western populations, and the morbidity and mortality associated with diabetes are becoming common in young Asians [2,3]. Type 2 diabetes has also become an important public health challenge among ethnic Chinese populations, especially in Taiwan, mainland China, Hong Kong, and Singapore, which comprise at least one-fifth of the global population [4-7]. In Taiwan, the prevalence rates of diabetes established from 1985 to 1996 were between 4.9% and 9.2% [4], which increased with age and varied with gender, race, and ethnicity. The Nutrition and Health Survey in Taiwan showed that the estimated prevalence rates of type 2 diabetes among the 19–44, 45–64, and ≥ 65 age groups (in years) were 0.6%, 11.4%, and 22.0%, respectively, among females and 1.1%, 7.0%, 7.2%, respectively, among males from 1993 to 1996 [8]. Furthermore, the estimated overall diabetes prevalence rates were 12.0% among men and 8.0% among women from 2005 to 2008 [9].

Previous studies estimating the incidence of type 2 diabetes in Taiwan are few. Recent studies have estimated that the overall five-year incidence among men and women in Taiwan was 187.1 and 218.4, respectively, per 100,000 people from 1992 to 1996 [10]. From 1999 to 2004, the overall five-year incidence remained stable at 7.6 among men and 7.7–6.9 among women per 1,000 person-years [11]. Furthermore, a previous study estimated the incidence of type 2 diabetes in persons aged ≥20 years and exempted from paying the National Health Insurance (NHI) premium was 20.4 per 1,000 person-years [12]. Thus, contemporary data are needed to update the existing demographics of diabetes in Taiwan.

Most prevalence estimates in Taiwan are based on relatively small populations with limited nationwide representativeness. The estimates reflect conditions at least 10 years ago or originate from studies using various methods to ascertain type 2 diabetes. Although the estimates of incidence in Taiwan are based on large samples, new data are needed to obtain information on existing conditions. In addition, time trend analyses of the annual prevalence and incidence of type 2 diabetes for a longer period using standardized methods have never been reported. The present study aims to conduct time trend analyses of the prevalence and incidence of type 2 diabetes in Taiwan from 2000 to 2007 in the population aged ≥ 20 years and enrolled in the Taiwan NHI program.

## Methods

### Data sources

The Taiwanese government launched the NHI program in March 1995, and 22.60 million out of the 22.96 million total population were enrolled in this program in 2007 [13]. After the implementation of the NHI program in 1995, about 98% of Taiwan’s population was covered. By the end of 2008, more than 99% was covered by the NHI program [14]. The NHI Bureau had contracts with 97% of hospitals and 92% of clinics all over the nation [15]. Expert reviews of a random sample of every 50–100 ambulatory and inpatient claims in each hospital and clinic were conducted quarterly to enhance the validity of the claims data. A severe penalty is imposed by the NHI Bureau on every false diagnosis report. The current study used the 1996 to 2007 registry datasets for beneficiaries, outpatient care by visits, and inpatient care by admission. Every individual in Taiwan has a unique personal identification number (PIN). The data on patient identities were scrambled cryptographically by the National Health Insurance Research Database (NHIRD) to protect patient privacy. All NHI datasets can be interlinked with the PIN of each individual. Registry files for beneficiaries were used to define the population for each year. The exclusion criteria discounted individuals who were not insured and who were younger than 20 years. The study population for each specific year was defined. These datasets are publicly available and were released by the institutes of the Taiwanese government.

### Ascertainment of diagnosed, and incident and prevalent cases

The population of patients diagnosed with type 2 diabetes was identified from diabetes datasets that contain original claims data for outpatient visits and inpatient admissions. Claims datasets from 2000 to 2007 were searched to identify any outpatient visit or inpatient admission with diabetes as one of the diagnoses (International Classification of Diseases, 9th Revision, Clinical Modification, ICD-9-CM code 250). Patients were classified as having type 2 diabetes and were thus included in the analysis if they had at least three ambulatory claims with a diagnosis of ICD-9-CM code 250 or A-code A181, or at least one inpatient claim with diabetes as one of the discharge diagnoses (ICD-9-CM code 250) during the specific year period. Patients who were type 2 diabetic but younger than 20 years, who were in gestation (ICD-9 code 6480), or who were type 1 diabetic (ICD code 250.×1, 250.×3) were excluded from this study.

An incident case was ascertained after confirming that the patient did not meet the criteria of type 2 diabetes in the claims datasets of the specific calendar year prior to his/her diabetes diagnosis. The claims datasets contained all types of health care services since 1996. Thus, the incident cases identified after 1 January 2000 had at least a four-year diabetes-free observation period. Incident cases were ascertained until 31 December 2007. The hospital admission date or the date of the first outpatient visit that met the definition for diabetes, whichever came first, was used as the date of incident event. Patients remained as prevalent cases regardless of whether or not they had diabetes diagnosis claims in the subsequent calendar year, as long as they remained in the datasets.

### Socio-demographic and urbanization levels of residential area

The socio-demographic factors studied included age, gender, and insurance premium. Age was categorized into four levels: 20–39, 40–59, 60–79, and ≥ 80 (in years). Insurance premiums were categorized into three levels: dependent population based on insurance premium, insurance premium less than its median value, and insurance premium greater than or equal to its median value. The dependent population consisted of individuals not in the labor force, such as students, housewives, and retirees. The insurance premium of an individual was determined by his/her work salary. The median values of the insurance premium were 19,400, 19,200, 16,500, and 21,000 Taiwan dollars in 2000, 2001, and 2003–2006, 2002, and 2007, respectively.

The residential areas of the study subjects, which covered 365 Taiwan townships, were classified into seven levels of urbanization according to the method developed by Liu et al. [16]. The seven levels of urbanization were high-density urban area, medium-density urban area, newly developed area, general area, aging society area, rural area, and non-developed area (seclusion area). The variables used in developing the township stratification for urbanization level consisted of the population density (people/km^2^), population ratio of people with an educational level of college or above, population ratio of elder people over 65 years old, population ratio of agricultural workers, and the number of physicians per 100,000 people, among others.

### Statistical analysis

The prevalence and incidence rates of patients clinically diagnosed with type 2 diabetes from 2000 to 2007 were estimated. The estimates of the annual prevalence rates according to age, gender, insurance premium, and urbanization level were obtained by dividing the number of prevalent cases of diagnosed type 2 diabetes identified in the NHI datasets by the total number of residents enrolled in the NHI program in a given year. The annual incidence rates of clinically diagnosed type 2 diabetes were estimated according to socio-demographic category by dividing the number of newly diagnosed type 2 diabetes cases by the total number of insured individuals who did not have type 2 diabetes at the beginning of the year in the NHI program. Annual incidence was expressed as per 1,000 residents of the source population in a given year.

The prevalence and incidence rates of diagnosed type 2 diabetes were adjusted using a direct standardization method that employs the age- and gender-specific rates of each year and age and gender distributions of the study population for the year 2000. The weights for the 20–39, 40–59, 60–79, and ≥ 80 age groups were 20.93%, 19.44%, 8.84%, and 1.17%, respectively, for men and 21.43%, 19.32%, 7.65%, and 1.21%, respectively, for women.

Multivariate logistic regression models were used to analyze the trends in prevalence and incidence over time while controlling the changes in the underlying age, sex, insurance premium, and urbanization level distributions. The dependent variable was diagnosed diabetes prevalence/incidence (diabetes = 1; non-diabetes = 0), and the categorical predictor variables were entered for the year (2000 as the reference), age group (20–39 years as the reference), gender (female as the reference), insurance premium (dependent population as the reference), and urbanization level (high-density urban area as the reference). If a time trend was observed, the rates of change in prevalence were analyzed by replacing the set of categorical variables for the calendar year with a continuous variable defined as time (in years). The terms of the age group variable multiplied by the time variable were added as covariates to examine the effects of the interaction between age group and time, as well as to investigate whether the rates of change over time in prevalence or incidence differed across age groups. A statistically significant interaction between time and a given age group indicated that the rate of change in prevalence/incidence differed over time compared with the reference age group (60 to 69 years).

## Results

A total of 1,557,494 patients in the NHIRD were identified as prevalent diagnosed cases from 2000 to 2007. The mean age of the prevalent diagnosed cases was 62.6, with a standard deviation (SD) of 13.3. The source population for which the prevalence was estimated was 12,191,076 in 2000 and 18,772,180 in 2007.

A 43.4% increase (from 5.8% to 8.3%) in the crude annual prevalence of diagnosed type 2 diabetes occurred from 2000 to 2007 (Table 1). After the direct standardization of the population in 2000, the annual standardized prevalence rates, as well as the discrepancy between crude and standardized annual prevalence rates, increased over time. Higher annual prevalence rates were observed in the older age, dependent population, and aging society area groups. The annual prevalence rates increased from 5.9% to 8.0% among women and from 5.7% to 8.6% among men. The rates were higher among females before 2002, but the numbers decreased thereafter.

**Table 1:** Prevalence and incidence of type 2 diabetes in Taiwan

	**2000**	**2001**	**2002**	**2003**	**2004**	**2005**	**2006**	**2007**
**Prevalence rates of type 2 diabetes**								
**Prevalent cases**	706,444	795,650	906,794	1,045,892	1,166,113	1,294,594	1,421,940	1557494
**Age (year)**	62.1±12.8	62.1±12.9	62.1±13.0	62.0±13.2	62.3±13.3	62.8±13.6	63.2±13.7	63.7±13.9
**Total population**	12,191,076	13,122,056	14,543,692	16,797,716	17,284,273	17,891,631	18,327,156	18,772,180
**Prevalence rate (%)**	5.8	6.1	6.2	6.2	6.8	7.2	7.8	8.3
**Standardized prevalence rate (%)**	5.8	6.2	6.5	6.8	7.3	7.8	8.2	8.5
**Age**								
20–39	0.6	0.6	0.6	0.6	0.7	0.7	0.8	0.8
40–59	5.6	6.0	6.3	6.5	7.0	7.3	7.7	8.0
60–79	17.6	18.7	19.8	20.8	22.2	23.6	24.8	25.9
≥80	16.4	18.0	19.6	21.2	23.3	25.4	27.2	28.9
**Sex**								
Female	5.9	6.1	6.2	6.1	6.6	7.0	7.5	8.0
Male	5.7	6.1	6.3	6.4	6.9	7.4	8.0	8.6
**Insurance premium**								
Dependent population	8.3	8.5	8.8	8.9	9.5	10.5	11.5	12.5
< Median	5.3	6.8	5.4	6.8	7.4	7.8	8.3	8.6
≧Median	5.2	3.5	5.7	3.5	3.8	4.0	4.2	4.3
**Urbanization level**								
High-density urban area	5.1	5.3	5.5	5.4	5.9	6.3	6.8	7.3
Medium-density urban area	5.8	6.0	6.2	6.2	6.7	7.2	7.7	8.3
Newly developed area	5.4	5.6	5.8	5.8	6.2	6.7	7.1	7.6
General area	6.6	7.0	7.2	7.4	8.0	8.6	9.2	9.8
Aging society area	8.6	9.1	9.5	10.0	10.8	11.8	12.7	13.6
Rural area	7.4	7.9	8.3	8.7	9.6	10.5	11.4	12.3
Non-developed area	6.9	7.4	7.7	8.0	8.8	9.5	10.2	11.0
**Incidence rates of type 2 diabetes**								
**Incident cases**	94,895	99,571	108,261	112,737	136,891	128,702	129,702	136,914
**Age (year)**	59.5±13.7	59.2±13.7	59.1±13.8	58.8±13.9	58.2±14.1	58.5±14.2	58.2±14.1	58.3±14.0
**Population at risk**	10,563,201	11,151,246	12,075,839	13,574,064	15,858,527	16,379,127	16,807,297	17,147,703
**Incidence rate (/1000)**	9.0	8.9	9.0	8.3	8.6	7.9	7.7	8.0
**Standardized incidence rate (/1000)**	9.0	9.1	9.3	8.9	9.8	8.9	8.7	8.9
**Age**								
20–39	1.5	1.5	1.5	1.4	1.5	1.4	1.4	1.4
40–59	9.8	10.1	10.3	9.9	11.0	9.9	9.9	10.3
60–79	23.9	23.8	24.5	23.5	25.5	23.7	22.8	23.3
≥80	25.1	24.8	25.5	24.6	25.7	22.9	20.0	19.2
**Sex**								
Female	8.6	8.4	8.3	7.6	7.9	7.2	7.0	7.3
Male	9.4	9.5	9.7	9.0	9.4	8.6	8.4	8.7
**Insurance premium**								
Dependent population	11.3	11.0	11.1	10.4	11.1	10.3	10.1	10.5
< Median	8.7	10.0	8.2	9.2	9.4	8.5	8.3	8.4
≥Median	8.4	6.1	8.5	5.6	5.8	5.3	5.3	5.5
**Urbanization level**								
High-density urban area	7.9	8.0	8.0	7.3	7.8	7.1	7.0	7.2
Medium-density urban area	9.0	8.9	9.0	8.2	8.6	7.8	7.8	8.0
Newly developed area	8.3	8.3	8.4	7.9	8.1	7.3	7.1	7.4
General area	10.2	10.0	10.1	9.6	9.9	8.9	8.7	9.2
Aging society area	12.9	13.1	12.9	12.6	12.9	12.1	12.1	12.5
Rural area	11.4	11.4	11.6	10.8	12.1	11.5	11.1	11.6
Non-developed area	10.5	10.4	10.8	10.5	10.9	10.2	9.8	10.1

**Figure 1:**
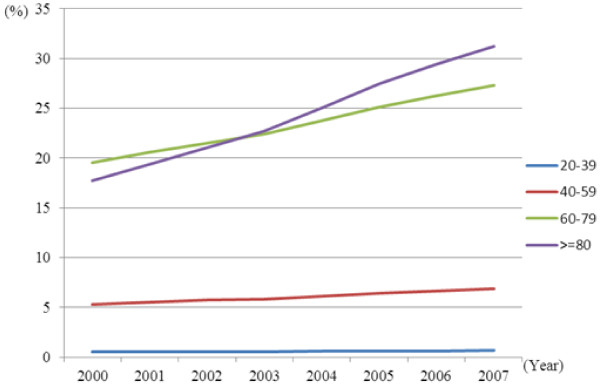
Time trends in the prevalence of type 2 diabetes stratified by age in females.

**Figure 2:**
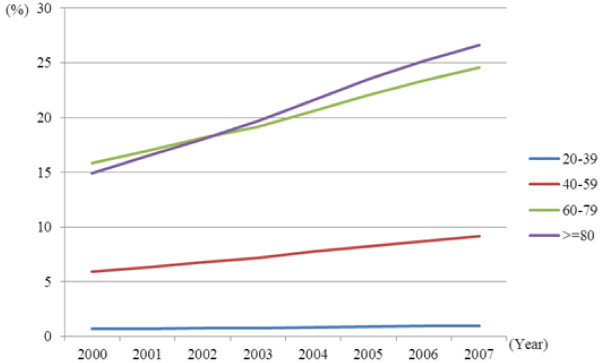
Time trends in the prevalence of type 2 diabetes stratified by age in males.

Significant interactions in the annual prevalence rates were observed between time and age groups for both genders (p < 0.05; Figures 1 and 2). The annual increase in diabetes prevalence rates were largest in the age group ≥ 80 years, followed by the 60–79, 40–59, and 20–39 age groups. From 2000 to 2007, annual prevalence increased by 76.7% (from 17.7% to 31.3%) among females and by 78.7% (from 14.9% to 26.6%) among males in the 80 years and over group; by 39.6% (from 19.5% to 27.3%) among females and 55.3% (from 15.8% to 24.6%) among males in the 60–79 group; by 29.4% (from 5.3% to 6.9%) among females and 55.4% (from 5.9% to 9.2%) among males in the 40–59 group; and by 26.4% (from 0.5% to 0.7%) among females and 47.0% (from 0.7% to 1.0%) among males in the 20–39 group.

The number of annual incident cases of diagnosed type 2 diabetes increased from 94,895 to 136,914 from 2000 to 2007, as well (Table 1). The mean age of incidence case patients was 58.7, with a SD of 13.9. The crude annual incidence fluctuated between 7.7 per 1,000 and 9.0 per 1,000 throughout the study duration. After the direct standardization of the Taiwanese population in 2000, the annual incidence rates gradually increased from 2000 to 2002, and then fluctuated thereafter.

After multivariate adjustment, the prevalence of type 2 diabetes was found to be significantly associated with age [OR (95% CI): 10.0 (10.0–10.0), 30.2 (31.7–31.9), and 31.8 (31.7–31.9) for the age groups 40–59, 60–79, and ≥ 80 years, respectively; Table 2]. Prevalence was generally high among males [1.0 (1.0–1.0)] and among individuals living in medium-density urban areas [1.1 (1.1–1.1)], newly developed areas [1.0 (1.0–1.0)], general areas [1.0 (1.0–1.0)], aging society areas [1.1 (1.1–1.1)], rural areas [1.0 (1.0–1.0)], and non-developed areas [1.1 (1.1–1.1)]. However, prevalence was lower among individuals with an insurance premium < median [0.7 (0.7–0.7)] and ≥ median [0.8 (0.8–0.8)]. The annual incidence rate fluctuated instead of following a linear trend. Thus, considering time as an ordinal variable, the annual incidence rate was not significant. The incidence rates among males were higher [1.2 (1.1–1.2)], especially among the 60–79 [15.5 (15.3–15.6)], middle insurance premium [0.9 (0.9–0.9)], and aging society area [1.1 (1.1–1.1)] groups (Table 3).

**Table 2:** Multivariate-adjusted relative risk of annual prevalence of type 2 diabetes rates for sex, age, time, insurance premium, and urbanization level

**Variable**	**Crude relative risk (95% CI)**	**Multivariate-adjusted relative risk (95% CI)a**
**Logistic regression model-type 2 diabetes prevalence**		
2000	1	1
2001	1.1 (1.0–1.1)***	1.0 (1.0–1.0)***
2002	1.1 (1.1–1.1)***	1.1 (1.1–1.1)***
2003	1.1 (1.1–1.1)***	1.1 (1.1–1.1)***
2004	1.2 (1.2–1.2)***	1.2 (1.2–1.2)***
2005	1.3 (1.3–1.3)***	1.3 (1.3–1.3)***
2006	1.4 (1.3–1.4)***	1.4 (1.4–1.4)***
2007	1.4 (1.4–1.4)***	1.4 (1.4–1.4)***
**Sex**		
Male	1.1 (1.0–1.1)***	1.0 (1.0–1.0)***
Female	1	1
**Age**		
20–39	1	1
40–59	9.9 (9.9–9.9)***	10.0 (10.0–10.0)***
60–79	31.5 (31.4–31.6)***	30.2 (30.1–30.3)***
≥ 80	34.1 (34.0–34.2)***	31.8 (31.7–31.9)***
**Insurance premium**		
Dependent population	1	1
<Median	0.7 (0.7–0.7)***	0. 9 (0.9–0.9)***
≥Median	0.4 (0.4–0.4)***	0.8 (0.8–0.8)***
**Urbanization level**		
High-density urban area	1	1
Medium-density urban area	1.1 (1.135–1.139)***	1.1 (1.1–1.1)***
Newly developed area	1.0 (1.046–1.050)***	1.0 (1.0–1.0)***
General area	1.3 (1.330–1.336)***	1.0 (1.0–1.0)***
Aging society area	1.8 (1.8–1.8)***	1.1 (1.1–1.1)***
Rural area	1. 6 (1.6–1.6)***	1.0 (1.0–1.0)***
Non-developed area	1.4 (1.4–1.5)***	1.0 (1.0–1.1)***

***: p < 0.001.

a: Modeled as an ordinal variable in a separate multivariate model with insurance premium, sex, age, insurance premium, and urbanization degree.

CI: confidence interval.

**Table 3:** Multivariate-adjusted relative risk of annual incidence rates of type 2 diabetes for sex, age, time, insurance premium, and urbanization level

**Variable**	**Crude relative risk (95% CI)**	**Multivariate-adjusted relative risk (95% CI)**
**Logistic regression model-type 2 diabetes incidence**		
2000	1	1
2001	1.0 (1.0–1.0)	0.9 (0.9–1.0)***
2002	1.0 (1.0–1.0)	1.0 (1.0–1.0)***
2003	0.9 (0.9–0.9)***	0.9 (0.9–1.0)***
2004	0.9 (0.9–0.9)***	1.0 (1.0–1.1)***
2005	0.9 (0.9–0.9)***	0.9 (0.9–0.9)***
2006	0.9 (0.9–0.9)***	0.9 (0.9–0.9)***
2007	0.9 (0.9–0.9)***	0.9 (0.9–0.9)***
**Sex**		
Male	1.2 (1.2–1. 2)***	1.2 (1.1–1.2)***
Female	1	1
**Age**		
20–39	1	1
40–59	6.9 (6.9–7.0)***	7.0 (6.9–7.1)***
60–79	16.2 (16.1–16.3)***	15.5 (15.3–15. 6)***
≥80	15.6 (15.4–15.8)***	14.9 (14.7–15.0)***
**Insurance premium**		
Dependent population	1	1
<Median	0.8 (0.8–0.8)***	0.9 (0.9–0.9)***
≥Median	0.6 (0.6–0.6)***	0.8 (0.8–0.8)***
**Urbanization level**		
High-density urban area	1	1
Medium-density urban area	1.1 (1.1–1.1)***	1.1 (1.1–1.1)***
Newly developed area	1.0 (1.0–1.0)***	1.0 (1.0–1.0)***
General area	1.3 (1.3–1.3)***	1.0 (1.0–1.1)***
Aging society area	1.7 (1.7–1.7)***	1.1 (1.1–1.1)***
Rural area	1.5 (1.5–1.5)***	1.1 (1.1–1.1)***
Non-developed area	1.4 (1.4–1.4)***	1.1 (1.1–1.1)***

***: p < 0.001.

CI: confidence interval.

## Discussion

The current study used the nationwide NHI claims data to estimate the annual prevalence and incidence of type 2 diabetes diagnosed from 2000 to 2007. Increases in the prevalence rate were observed in both crude age- and gender-standardized annual prevalence rates from 2000 to 2007. The annual standardized incidence rates fluctuated throughout the period.

The results showed that the annual prevalence of type 2 diabetes continued to rise throughout the study period. The World Health Organization (WHO) predicted that a 39% increase in the global prevalence of diabetes would occur between 2000 and 2030. However, a 43.35% increase in prevalence has already occurred over an eight-year period in Taiwan. During the same period, the crude incidence rate did not exhibit a linear growth trend. Instead, it decreased slightly since 2005. Thus, the increase in the prevalence of type 2 diabetes may be partly explained by better diabetes care and longer survival.

The cross-sectional studies in various areas of Taiwan included the Ann-Lo district in northern Taiwan in 1988–1990 [17], the Kin-Hu and Kinmen offshore islands in 1991–1994 [18], Pu-li township in central Taiwan in 1987–1988 and 1991–1992 [19], Tainan city in southern Taiwan in 1996 [18], and the Penghu offshore islands in 1995–1997 [20] with a prevalence of 5.6%–9.0%. All these studies were based on small samples collected in restricted areas; thus, their estimates may not be reliable and valid for national estimates. In addition, these studies were conducted before 2000. After 2000, Chang et al. [11] used the NHI database, which consisted of 15 million individuals from 23 million insured people registered in the NHI program of Taiwan, to estimate the prevalence of type 2 diabetes. The current study made use of 23 million insured people under the NHI program. The estimated prevalence and incidence using the same definition of type 2 diabetes and datasets were found to be similar to those of Chang et al. [11]. The present study is more reliable and provides more precise prevalence and incidence estimates because of the larger sample size. Furthermore, this study provides prevalence and incidence rates across several socio-demographic subgroups. Compared with the prevalence rates for the same year, the prevalence based on our findings is lower than those for India (12.1% in 2000), Sri Lanka (10.3% in 2006), and Korea (7.6% in 2001); closer to those for China (6.1% in 2002), Thailand (6.7% in 2004), and the Philippines (6.5% in 2004); and higher than that for Vietnam (3.8% in 2001) [2,6,7].

Previous studies have shown that there is an increasing trend in the prevalence of type 2 diabetes globally; specifically, prevalence increased by 28.3% among men and 25.9% among women in Sweden from 1972 to 2001 [21], 48% in America from 1990 to 1998 [22], 47% in England from 1994 to 2001 [23], 69% in Canada from 1995 to 2005 [24], and 38% among men and 25% among women in Taiwan from 1999 to 2004 [11]. The findings in the present study further demonstrated that the prevalence of type 2 diabetes also increased from 2005 to 2007, with a greater increase among men, which was consistent with the findings of previous studies [21,23]. Previous studies examining the incidence trend of type 2 diabetes in Taiwan showed that the incidence rates among both men and women increased from 1992 to 1996 in Taiwan [10]. However, the incidence rates remained stable among men and slightly decreased among women from 1999 to 2004, after considering age and gender [11]. Thus, the trend toward a decreasing incidence among women and the stable incidence rate among men may be the reason for the higher prevalence among men than women that was observed after 2002.

The initial higher prevalence rates among females before 2002 and the subsequent decrease were supported by a previous study, which compared two nutrition and health surveys from 1993–1996 and 2005–2008 [9]. The prevalence of diabetes in women was 5.5% in 1993–1996 and 8.0% in 2005–2008, whereas that in men was 3.2% in 1993–1996 and 12.0% in 2005–2008. This trend may be explained by the increased prevalence of obesity in men, which saw an increase from 33.4% in 1993–1996 to 51.0% in 2005–2008; however, obesity among women remained relatively stable, increasing from 33.5% in 1993–1996 to 35.9% in 2005–2008. The increased consumption of cakes, sweets, and sugary drinks, as well as the increased proliferation of a sedentary lifestyle, may have lead to this increase in obesity [9].

Of note, a decline in type 2 diabetes prevalence among individuals with an insurance premium greater than or equal to a median value over the period of observation was observed, which is consistent with the data reported by the International Diabetes Federation [25]. This socio-economically advantaged subgroup also experienced the greatest decline in incidence rate, which is similar to the findings reported by Hsu et al. [12], who indicated that poverty is associated with higher diabetes incidence.

Increasing levels of obesity in the general population in the past two decades are believed to be one of the principal factors contributing to the rising incidence of type 2 diabetes in Asia [26,27]. The Department of Health in Taiwan has implemented many health promotion programs in communities and workplaces in the past 10 years for the prevention of chronic diseases [15]. The major component of these promotion programs is an increase in the level of physical activity of residents in Taiwan. The protective effects of physical activities include improved body composition, glucose tolerance, and insulin sensitivity [28,29], and this may have contributed to the decline in the incidence of type 2 diabetes since 2004, as determined in the present study.

The present study has four strengths. First, nationwide data with a large sample size was used, which emphasized the prevalence and incidence trends with a standardized method to define type 2 diabetes. Second, the NHI program in Taiwan provides continuing universal coverage for the entire population, which avoids selection bias. Third, NHI datasets were used, which eliminated the need to minimize the numbers of subjects in the cohorts lost to follow-up. In addition, a large sample of geographically dispersed patients was easily obtained. Finally, a large number of study subjects facilitated the age- and sex-stratified analyses with an ample sample size to satisfy requirements.

Nevertheless, the current study also has several limitations. First, some cases of type 1 diabetes may have been falsely classified as type 2 diabetes. Diabetic patients aged 20 years and over were included, and individuals with type 1 diabetes, as defined by the ICD-9 code, were excluded to minimize the misclassification. Second, the study depended exclusively on claims data, which may have resulted in potential intentional or unintentional disease misclassification bias. Patients with at least three ambulatory claims or at least one inpatient claim with a diagnosis of type 2 diabetes were included during the specific year period to minimize potential misclassification. Thus, the estimates of prevalence and incidence may be underestimated. Finally, the body mass index, waist circumference, blood pressure, smoking history, family history of diseases, and laboratory test results were not available in the claims database. Thus, combining this information with the discussion on the prevalence and incidence of type 2 diabetes was beyond the scope of this study.

Diabetes is associated with an increased risk of co-morbidity, such as cancer [30], dementia [31], and Parkinson’s disease [32]. Previous studies indicated that the cancer incidence density increases by 2.12, 2.80, 4.13, 2.10, and 2.58-fold for total cancer, colorectal cancer, hepatocellular cancer, pancreatic cancer, and dementia, respectively, for diabetes patients without anti-hyperglycemic medication [30,31]. Considering the time trend of the prevalence of type 2 diabetes indicated in our study, we would estimate that the number of incident cases of total cancer, colorectal cancer, hepatocellular cancer, pancreatic cancer, and dementia would increase by 4391, 1083, 1568, 229, and 6196, respectively, based on the risk difference between individuals without diabetes and those with diabetes but no anti-hyperglycemic medication, as reported by Lee et al. [30] and Hsu et al. [31]. Cancer and depression prevention may be enhanced by the primary prevention of diabetes mellitus or diabetes mellitus medication [30-32].

## Conclusion

The NHI claims data used in this study showed that the annual prevalence of type 2 diabetes increased in Taiwan from 2000 to 2007, whereas the annual standardized incidence fluctuated throughout the study period. Public health professionals are recommended to plan appropriate health promotion programs, such as exercise and a healthy diet, for men, individuals aged 40 years and over, and residents of areas with lower levels of urbanization.

## Competing interests

The authors declared that they have no competing interest.

## Authors’ contributions

TCL and CCLin contributed equally to the design of the study and the direction of its implementation, including supervision of the field activities, quality assurance and control. CIL, CSL, and CCLee supervised the field activities. CSL, CCLin and CIL helped conduct the literature review and prepare the Methods and the Discussion sections of the text. CYH, CIL, TCL and SYY designed the study’s analytic strategy and conducted the data analysis. All authors read and approved the final manuscript.

## Pre-publication history

The pre-publication history for this paper can be accessed here:

http://www.biomedcentral.com/1471-2458/13/318/prepub
